# Evaluation and Immunolocalization of BMP4 and FGF8 in Odontogenic Cyst and Tumors

**DOI:** 10.1155/2018/1204549

**Published:** 2018-07-12

**Authors:** Neeti Swarup, Meghanand T. Nayak, Zoya Chowdhary, Monica Mehendiratta, Shikha Khatana, Su Jung Choi, Chandarani Sagolsem

**Affiliations:** ^1^Department of Oral Pathology and Microbiology, Daswani Dental College and Research Centre, Kota, India; ^2^Department of Oral Pathology and Microbiology, Teerthanker Mahaveer Dental College and Research Centre, Moradabad, India; ^3^Department of Periodontology, Indira Gandhi Government Dental College and Hospital, Jammu, India; ^4^Department of Oral Pathology and Microbiology, ITS Dental College, Greater Noida, India; ^5^Department of Oral Pathology and Microbiology, Sudha Rustagi College of Dental Sciences, Faridabad, India; ^6^Department of Oral Pathology, School of Dentistry, Seoul National University, Seoul, Republic of Korea; ^7^Department of Conservative Dentistry and Endodontics, Daswani Dental College and Research Centre, Kota, India

## Abstract

Growth factors like bone morphogenetic protein 4 (BMP4) and fibroblast growth factor 8 (FGF8) play a major role in organogenesis and specifically in odontogenesis. They are also believed to have a role in oncogenesis. Thus, any discrepancies in their standard behavior and activity would lead to serious abnormalities including odontogenic cyst and tumors. The present research work investigated the expression of BMP4 and FGF8 in odontogenic tumors (OT) and cyst as well as developing tooth germs to elucidate their roles. Dental organs of various odontogenic stages and 30 OTs including solid multicystic ameloblastomas (SMA, 10 cases), ameloblastic fibroma (AF, 10 cases), odontogenic myxoma (OM, 10 cases), and odontogenic cysts: odontogenic keratocyst (OKC, 10 cases) were evaluated in both epithelial and mesenchymal components for the expression of BMP4 and FGF8 using immunohistochemistry. The epithelial nuclear expression of BMP4 was highest in OKC (9 cases) while FGF8 was highest in SMA (10 cases). The mesenchymal nuclear expression of both BMP4 (8 cases) (*p* = 0.001) and FGF8 (9 cases) (*p* = 0.045) were significantly high in OMs among all OTs. Both growth factors were actively expressed in different stages of tooth development. The expression of BMP4 and FGF8 corelates well with the proliferative component of the pathologies, indicating a possible role in the pathogenesis and progression.

## 1. Introduction

Odontogenic tumors (OTs) are a unique group of neoplasms, derived from tooth forming apparatus or its remnants, hence found exclusively in the jaws or associated soft tissue [1]. In 2005, World Health Organization (WHO) classified them broadly under benign and malignant and subclassified them further based on tissue of origin, that is, epithelial, ectomesenchymal, or both; this however led to inclusion and classification of odontogenic keratocyst (OKC) as keratocystic odontogenic tumor in group 1 tumors [2].

Recently, the status OKC was addressed and classified into the category of cyst. Based on WHO histological classification of OTs and odontogenic cysts (2017), three benign tumors were considered for the study, namely, solid multicystic ameloblastoma (SMA) from group1 (epithelial origin); ameloblastic fibroma (AF) from group 2 (mixed origin); and odontogenic myxoma (OM) from group 3 (ectomesenchymal origin) and OKC was included in the study owing to its aggressive nature [3].

The pathogenesis of OTs like any tumor is dependent on two factors, tumor initiating factors and tumor progression factors. Genes expressing these factors in OTs show a striking resemblance to those expressed during odontogenesis [1]. OKC arises from remnants of the dental lamina (rest of Serres) due to inactivation of PTCH1, which activates Shh pathway leading to excessive proliferation [3]. Odontogenesis is a very complex process monitored by intricate interactions between homeobox genes and their signaling molecules; sonic hedgehog (Shh), Wnt, BMP, and FGF [4].

Bone morphogenetic proteins (BMP) is a member of transforming growth factor (TGF *β*) superfamily. There are more than 30 known types of BMP and are well known as the signaling molecule, which plays a crucial role in cellular proliferation, extracellular matrix production along with differentiation of neoplastic tissues [5]. BMP4 plays a key role in Msx-1-dependent mesenchymal odontogenic signaling for tooth morphogenesis, and it is also linked to epidermal differentiation [6, 7].

Fibroblast growth factors (FGF) perform a variety of cellular processes such as stemness, proliferation, and antiapoptosis. There are 18 members which are grouped under 6 subfamilies along with four receptors [8]. FGF8 plays an important role during embryogenesis and is required during the initiation of odontogenesis [9].

Odontogenic pathologies present with a wide array of molecular variations. The two described factors have been scarcely studied in OTs and OKC. The study of these two factors can lead to a better understanding of the behavior of OTs and OKC and pave the way for establishing better pathological evaluation processes and development of novel therapies. On this premise, we conducted the study to evaluate the expression of these two growth factors in OTs and OKC and furthermore compared with that of different stages of odontogenesis.

## 2. Materials and Methods

### 2.1. Specimen Collection

Forty cases of odontogenic pathologies, 10 cases for each SMA, OKC, AF, and OM, were retrieved from archives of six different institutes of North India: Teerthanker Mahaveer Dental College & Research Centre, Moradabad (*n* = 15); Institute of Dental Sciences, Bareilly (*n* = 12); Sudha Rustagi Dental College and Research Centre, Faridabad (*n* = 5); Subharti Dental College, Meerut (*n* = 4); Surendra Dental College & Research Centre, Sri Ganganagar (*n* = 2); and MedAid India, Noida (*n* = 2). Sections from mandibular tissue of chemically aborted fetus were obtained from Teerthanker Mahaveer Medical College and Research Centre to procure stages of odontogenesis: dental lamina, bud stage, cap stage, and bell stage. Three sections each of 3 microns thickness were obtained from formalin fixed paraffin embedded tissue. Hematoxylin and eosin stained section was used for diagnosis using WHO criteria (2017). Two sections were obtained on poly-L-lysine-coated slides and stained with BMP4 and FGF8 using immunohistochemical methods. Few additional sections of cases of epidermoid carcinoma and ductal carcinoma of breast were included to serve as controls for BMP4 and FGF8, respectively. The study was approved by the Institutional Ethical Committee, Teerthanker Mahaveer Dental College and Research Centre, Teerthanker Mahaveer University, Moradabad, U.P., India.

### 2.2. Immunohistochemistry

The deparaffinised sections on poly-L-lysine-coated slides after rehydration were subjected to the immunohistochemical procedure.

### 2.3. BMP4

The antibody clone used for detection of BMP4 was GTX100875 (Genetex Hsinchu City, Taiwan, polyclonal antibody). The antibody was diluted to a dilution of 1 : 100 by adding 1 *μ*l antibody to 99 *μ*l of Renoir Red Diluent®. Antigen retrieval was done by boiling the sections obtained on poly-L-ysine slides in Diva Decloaker antigen retrieval solution, BioCare DV2005 L2J (low pH 6.5), which was obtained by mixing 1 ml Diva Decloaker to 19 ml of distilled water in a pressure cooker at 120°C for 20–25 min. The sections were then incubated with antibody at room temperature in a humidity chamber for 1 hour and 15 minutes. For antibody detection, Leica Novolink™ Novocastra Polymer HRP Kit #RE 7290-CE was used as per directions. Epidermoid carcinoma was used as a positive control, and the negative control staining was performed without primary antibody.

### 2.4. FGF8

The antibody clone used for detection of FGF8 was PA1216 (Boster, Pleasanton, CA, polyclonal antibody). The lyophilised antibody was reconstituted by adding 0.2 ml distilled water to the 100 *μ*g antibody which yielded 500 *μ*g/ml. The reconstituted antibody was diluted to a dilution of 1 : 200 to yield a concentration of 0.5 *μ*g/ml in Renoir Red Diluent. Antigen retrieval was done by boiling the sections obtained on poly-L-lysine slides in Diva Decloaker antigen retrieval solution, BioCare DV2005 L2J (low pH 6.5), which was obtained by mixing 1 ml Diva Decloaker to 19 ml of distilled water in a pressure cooker at 120°C for 20–25 min. The sections were then incubated with antibody at room temperature in a humidity chamber for 1 hour and 15 minutes. For antibody detection, Leica Novolink Novocastra Polymer HRP Kit #RE 7290-CE was used as per directions. Ductal carcinoma of the breast was used as a positive control, and the negative control staining was performed without primary antibody.

The stained specimens were then sequentially examined by two observers under 4x, 10x and 40x magnification for BMP4 and FGF8 expression in the epithelial and mesenchymal component and were assessed for cytoplasmic or nuclear reactivity. A total of five high power fields were observed. The specimens were scored on the basis of the number of cells stained as described below.

Scoring criteria:

−/negative: no cells were stained.
2. +/positive: <20 percent cells were stained.
3. ++/strong positive: 20–80 percent cells were stained.
4. +++/very strong positive: >80 percent cells were stained.

### 2.5. Statistical Evaluation

The results obtained were subjected to statistical evaluation using SPSS version 21.0. Chi-square test was applied for statistical evaluation for intergroup comparison between OKC and OTs but due to smaller sample size for intragroup comparison between growth factors percentage was calculated for evaluation of data.

## 3. Results

**Figure 1:**
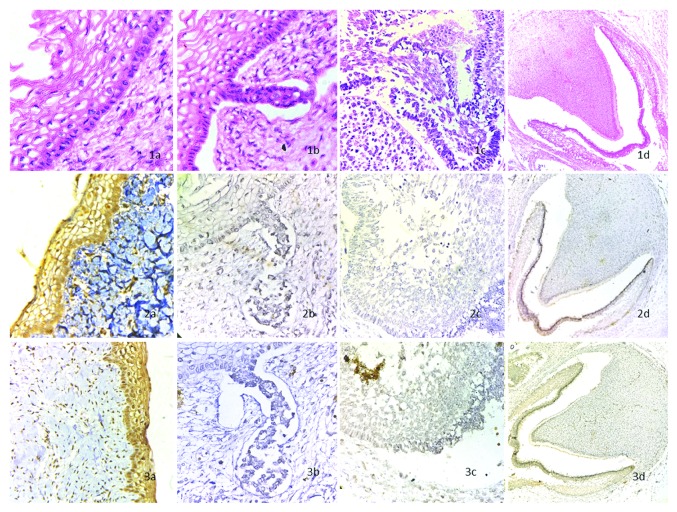
Staining of stages of odontogenesis: (1a) shows H and E stained dental lamina at x400; (1b) shows H and E stained bud stage at x400; (1c) shows H and E stained cap stage at x400; (1d) shows H and E stained bell stage at x100. (2a) shows BMP4 stained dental lamina at x400, the epithelium is strongly positive with moderate reactivity in ectomesenchyme; (2b) shows BMP4 stained bud stage at x400, the epithelium is nonreactive with slight reactivity in ectomesenchyme; (2c) shows BMP4 stained cap stage at x400, the epithelium and ectomesemchyme are nonreactive; (2d) shows BMP4 stained bell stage at x100, the epithelium and dental papilla is strongly positive. (3a) shows FGF8 stained dental lamina at x400, the epithelium is strongly positive with moderate reactivity in ectomesenchyme; (3b) shows FGF8 stained bud stage at x400, the epithelium and ectomesenchyme are nonreactive; (3c) shows FGF8 stained cap stage at x400, the epithelium and ectomesenchyme are nonreactive; (3d) shows FGF8 stained bell stage at x100, the epithelium and dental papilla is strongly positive.

**Figure 2:**
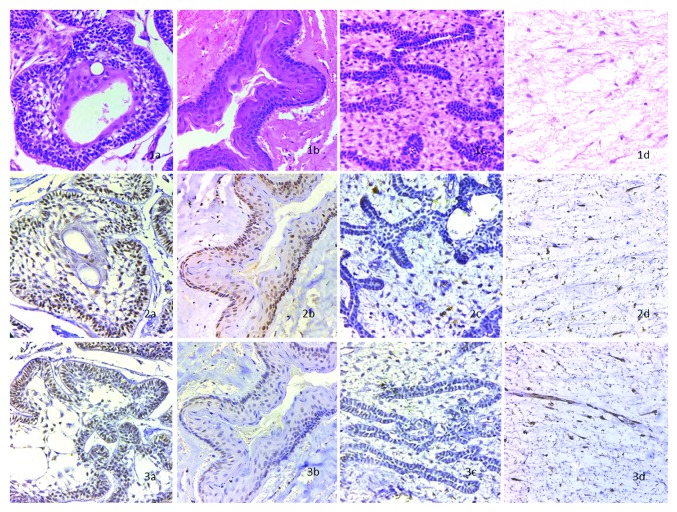
Staining of odontogenic tumors and odontogenic cyst: (1a) shows H and E stained SMA at x400; (1b) shows H and E stained OKC at x400; (1c) shows H and E stained AF at x400; (1d) shows H and E stained OM at x400. (2a) shows BMP4 stained SMA at x400, the epithelium is positive with slight reactivity in mesenchyme; (2b) shows BMP4 stained OKC at x400, the epithelium is strongly reactive with moderate reactivity in mesenchyme; (2c) shows BMP4 stained AF at x400, the epithelium and mesenchyme are mildly reactive; (2d) shows BMP4 stained OM at x400, the mesenchymal component is strongly positive. (3a) shows FGF8 stained SMA at x400, the epithelium is strongly positive with moderate reactivity in mesenchyme; (3b) shows FGF8 stained OKC at x400, the epithelium is positive and mesenchyme shows slight reactivity; (3c) shows FGF8 stained AF at x400, the epithelium and mesenchyme are mildly reactive; (3d) shows FGF8 stained OM at x400, the mesenchyme is strongly positive.

The stained specimens (Figures 1 and 2) were scored according to the criteria. The results obtained are summarized in Tables 1, 2, and 3.

### 3.1. Stages of Odontogenesis

Dental lamina demonstrated very strong cytoplasmic positivity for BMP4 and FGF8 in the ectodermal and ectomesenchymal components.

Bud stage dental organ demonstrated slight nuclear reactivity for BMP4 in the dental epithelium and weaker staining in the stroma. Reactivity for FGF8 was only found in the dental epithelium in both nucleus and cytoplasm.

The odontogenic epithelial components and ectomesenchymal components during the cap stage were reactive for FGF8 with mild nuclear and cytoplasmic positivity.

The bell stage dental organ showed a stronger cytoplasmic reactivity for both of these two factors in the epithelial and ectomesenchymal component.

### 3.2. Ameloblastoma

The epithelial component of all 10 out of 10 cases (100%) of SMA demonstrated nuclear localization of FGF8 but only 7 out of 10 cases (70%) were reactive to BMP4, whereas the mesenchyme was less reactive and demonstrated a similar result for both growth factors.

### 3.3. OKC

The nuclear localization of BMP4 was found in the epithelial component of 9 out of 10 cases (90%) of OKC whereas only 7 out of 10 cases (70%) demonstrated nuclear localization of FGF8, while the mesenchymal component was slightly more reactive for BMP4, 8 out of 10 cases (80%), as compared to FGF8, 6 out of 10 cases (60%).

### 3.4. Ameloblastic Fibroma

AFs showed a comparable nuclear and cytoplasmic expression in the epithelial component for BMP4, 7 out of 10 cases (70%), and FGF8, 6 cases out of 10 (60%). The mesenchymal component was slightly more reactive for BMP4, 6 out of 10 cases (60%).

### 3.5. Odontogenic Myxoma

The nuclear expression of FGF8, 9 out of 10 cases (90%), was stronger as compared to BMP4, 8 out of 10 cases (80%), whereas the cytoplasmic reactivity was comparable for both the growth factors.

### 3.6. BMP4 in OTs and OKC

The nuclear localization in the epithelial component was found to be highest in OKC (9 out of 10 cases, 90%), while the cytoplasmic reactivity was higher in AFs (5 out of 10 cases, 50%). The mesenchymal component of OMs (8 out of 10 cases, 80%) had highest nuclear reactivity for BMP4 (*p* = 0.001) while OKC (7 out of 10 cases, 70%) had greater cytoplasmic reactivity.

### 3.7. FGF8 in OTs and OKC

SMA (10 out of 10 cases, 100%) had the highest nuclear reactivity in the epithelial component for the same, while the cytoplasmic reactivity was higher in AFs (4 out of 10 cases, 40%). The mesenchymal component of OMs (9 out of 10 cases, 90%) had the highest nuclear reactivity for FGF8 (*p* = 0.045), while OKC (4 out of 10 cases, 40%) had greater cytoplasmic reactivity.

## 4. Discussion

Various components of the odontogenic apparatus can be identified as the source of origin for OTs and OKC. They may comprise of the odontogenic epithelium, the ectomesenchyme or may include both the components. The pathogenesis of OTs and OKC is a complex process, which at some stages resembles the odontogenic process. Odontogenesis is a highly regulated process monitored by regulatory genes and signaling factors. The regulatory genes belong to the homeobox family, and signaling factors belong to the BMP, FGF, Wnt, and Shh families [4].

BMPs are chief regulators of development, playing a major role in the organogenetic process, regenerative processes, and carcinogenetic process [5, 10]. By regulating target gene transcription, various cellular processes are controlled by these growth factors, such as proliferation, differentiation, apoptosis, migration, and angiogenesis. BMPs play a dual role in cancer cell regulation; they upregulate growth in certain tumors and downregulate growth in other tumors [10].

BMP4 is a member of BMP family and transforming growth factor beta-1 (TGF*β*1) superfamily of secretory signaling molecules that play vital roles in embryonic development [11]. It is a critical regulatory molecule performing a wide array of functions during development, including mesoderm induction, limb formation, tooth development, bone induction, and fracture repair.

BMP4 contributes to various cancer-associated phenotypes, including cell growth, differentiation, migration, invasion, and angiogenesis, which is critical for cancer development and progression. It has a dual role as a proapoptotic and antiapoptotic factor in different tumors. BMP4 is also an important regulator of cell migration and invasion and is known to induce the epithelial mesenchymal transition (EMT), which grants mobility to cancer cells and eventually aid in metastasis [12]. BMP FGF axis maintains proliferation of basal keratinocytes and plays a major role in epidermal stratification [7].

The family of FGFs regulates an ample number of developmental processes, including branching morphogenesis, brain patterning, and limb development [13, 14]. FGFs and their receptors control a wide range of biological functions, regulating cellular proliferation, survival, migration, and differentiation. Compagni et al. [15] indicated that FGFs play a crucial role in tumor angiogenesis. FGF8 plays a pivotal role during development and also during carcinogenesis.

FGF8 was originally referred to as androgen-induced growth factor (AIGF) as it played a role in the growth of androgen-dependent growth of mouse mammary tumor cell line [16]. FGF8 was found to impart proliferative and metastatic capacity of colorectal cancer cells [17]. It is also responsible for maintaining the progenitor status and fate determination of cranial neural crest cells [18]. Daphna-Iken et al. [19] demonstrated that production of FGF8b, and other FGF8 isoforms, contributed to oncogenesis in mammary and salivary glands, and that ovarian expression results in stromal hyperplasia.

In the present study, SMA showed stronger expression of FGF8 than BMP4 expression. FGF8 was found to induce odontogenic epithelium during the early stages of odontogenesis [9]. Zhong et al. [20] found that FGF8 overexpression in prostate epithelium led to prostate intraepithelial neoplasia. We may conclude that increased expression of FGF8 might play a role in tumor initiation and progression by inducing the odontogenic epithelium in cases of SMA. Expression of BMP4 in SMA was found to be in accordance with the study conducted by Kumamoto and Ooya [21]. There was a decreased expression of BMP4 in granular cell SMA in a study conducted by Sathi et al. [22] in 2007, but no cases of granular cell SMA were included in our study. Expression of BMP4 in cells with squamous metaplasia in the acanthomatous type of SMA was a distinctive finding.

In the present study, the BMP4 expression was stronger than FGF8 in cases of OKC. Increased expression of BMP4 in OKC was found to be in accordance with the study conducted by Kim et al. [23], but they were contrary to the results by Gao et al. [24], which indirectly concluded the downregulation of BMP4 signaling due to decreased expression of Smad4. BMP4 was found to be responsible for proliferative abilities of basal cell keratinocytes [7]. The study conducted by Heikinheimo et al. [25] suggests that SMA displayed an odontogenic fate while OKC displayed squamous epithelial fate. The preferential expression of FGF8 for SMA and BMP4 in OKC in our study may well explain the odontogenic and squamous epithelial fates, respectively.

The results obtained in our study suggest that the expression of BMP4 and FGF8 were equivalent in AF with slightly more cytoplasmic reactivity, which resembled the expression of BMP4 and FGF8 in the bell stage of odontogenesis. The milder expression and resemblance to the mature stage of tooth development may explain the indolent behavior of this tumor and more differentiated/mature neoplastic cells. Similar conclusions were drawn from the results obtained by So et al. [26], who revealed a comparable expression of FGF2 in AF and ameloblastic fibroodontoma and histodifferentiation stage of odontogenesis.

The expression of FGF8 and BMP4 were comparable with slightly more reactivity for FGF8 in OMs. Immunohistochemical studies for OMs regarding the two or related growth factors have been very scarce. Based on results of the study conducted by Molina et al. [27], expression of VEGF-A and ORM-1 may be associated with angiogenesis and tumor structural viscosity which may influence tumor growth in OMs. However, the limited data on upregulation of expression of FGF8 in English literature search was found in myxoinflammatory fibroblastic sarcoma [28] and few other tumors [14, 17, 19]. FGF8 was also found to have a synergistic role with VEGF in prostate cancer [29]. The results indicate a possible relation between FGF8 and initiation, progression and its interaction with other factors in the pathogenesis of the OMs, which requires further substantiation. We may conclude that an increased expression of BMP4 and FGF8 in OM may be responsible for imparting it an aggressive nature.

Overall, the reactivity for BMP4 was seen to be the highest in OKC when considering both epithelial and mesenchymal component among all tumors, but the mesenchymal reactivity was highest in OM. The epithelial component of all SMA showed reactivity for FGF8, whereas the mesenchymal reactivity was highest in OMs. Among all tumors, the overall reactivity for FGF8 in AF was low when compared to other tumors, although AF had a significant epithelial positivity for cytoplasmic BMP4.

Our results for expression of BMP4 in SMA were contradictory to Gao et al. [30]; they concluded from their study that SMA was nonreactive for BMP McAb but positive for group 2 and group 3 OTs. They attributed this expression to its role in hard tissue formation; however, newer roles of BMP4 in tumorigenesis and development have been elucidated by other researchers and now the function of BMP4 is no longer confined to hard tissue formation [30]. The expression of BMP4 and FGF8 in SMA and OKC noted in the present study were in accordance with the study conducted by Pimentel [31] in 2015.

Both BMP4 and FGF8 were found to be positive in the epithelial and mesenchymal components of different tumors under varying degrees, suggesting that they are involved in epithelial and mesenchymal interactions via paracrine and autocrine mechanisms [12].

Studies demonstrate that BMP4 suppresses cell growth both in vitro and in vivo, and at the same time is able to induce migration, invasion, and EMT. The dual role makes it an interesting growth factor, which may explain the aggressive nature of tumors like OM and OKC [10]. Furthermore, expression of BMP4 by keratinizing components in OKC and acanthomatous SMA may indicate their role in squamous differentiation.

Overexpression of FGFs in the epithelium induces carcinogenesis through an autocrine signal loop, which may clarify the oncogenetic changes taking place in the odontogenic epithelial component leading to the pathogenesis of SMA.

The decreased reactivity of both factors in the case of AF, when compared to other odontogenic pathologies, may explain the indolent behavior of the tumor when compared to different tumors.

In conclusion, the results suggest that BMP4 and FGF8 are expressed in different odontogenic cyst and tumors with varying intensity based on their expected behavior. The greater expression for growth factors in aggressive pathologies may suggest an analogy to proliferative stages of odontogenesis. While, in pathologies with milder clinical course, a lesser expression for growth factors was observed which may be considered to parallel with different stages of odontogenesis. The results also indicate that BMP4 and FGF8 may play important roles in the pathogenesis of the odontogenic pathologies and furthermore regulate their clinical behavior, which requires further genetic and proteomic studies with larger sample sizes to better understand the participation of these elements in these pathologies.

## Figures and Tables

**Table 1:** Evaluation and immunohistochemical score of stages of odontogenesis.

	Odontogenesis								
		Dental lamina		Bud stage		Cap stage		Bell stage	
		BMP4	FGF8	BMP4	FGF8	BMP4	FGF8	BMP4	FGF8
Ectodermal components									
Basal cells	C	+++	+++	−	+	Ab	Ab	Ab	Ab
	N	+	+	+	+	Ab	Ab	Ab	Ab
Inner enamel epithelium	C	Ab	Ab	Ab	Ab	−	−	+++	+++
	N	Ab	Ab	Ab	Ab	+	+	+	+
Stratum intermedium	C	Ab	Ab	Ab	Ab	Ab	Ab	+++	+++
	N	Ab	Ab	Ab	Ab	Ab	Ab	+	+
Stellate reticulum	C	Ab	Ab	Ab	Ab	−	−	+++	+++
	N	Ab	Ab	Ab	Ab	+	+	+	+
Outer enamel epithelium	C	Ab	Ab	Ab	Ab	−	−	+++	+++
	N	Ab	Ab	Ab	Ab	+	+	+	+
Ectomesenchymal components									
Stromal component	C	++	++	−	−	Ab	Ab	Ab	Ab
	N	+	+	+	−	Ab	Ab	Ab	Ab
Dental papilla	C	Ab	Ab	Ab	Ab	−	+	++	++
	N	Ab	Ab	Ab	Ab	−	+	++	++
Dental sac	C	Ab	Ab	Ab	Ab	−	+	++	++
	N	Ab	Ab	Ab	Ab	−	+	+	+

C: cytoplasm; N: nucleus; Ab: absent.

**Table 2:** Evaluation and immunohistochemical score of odontogenic tumors and odontogenic cyst.

SMA		SMA 1		SMA 2		SMA 3		SMA 4		SMA 5		SMA 6		SMA 7		SMA 8		SMA 9		SMA 10	
Epithelial component		BMP4	FGF8	BMP4	FGF8	BMP4	FGF8	BMP4	FGF8	BMP4	FGF8	BMP4	FGF8	BMP4	FGF8	BMP4	FGF8	BMP4	FGF8	BMP4	FGF8
Columnar cells (ameloblast like)	C	+	−	−	−	−	−	−	−	−	−	−	−	−	+	−	−	−	−	−	−
	N	+++	+++	++	+	−	+++	+++	+++	+++	+	++	+++	+	++	−	+	−	+	++	+
Central cells (stellate reticulum like)	C	++	−	−	−	−	−	−	−	−	−	−	−	−	−	−	−	−	−	−	−
	N	+++	+++	++	+	−	++	+++	++	+++	−	++	+++	++	++	−	−	−	−	−	+
Mesenchymal component	C	++	+	−	−	−	+	+	+	−	−	−	−	−	−	−	−	−	−	−	−
	N	++	+	−	−	−	+	+	+	−	−	−	−	−	−	+	+	−	+	−	−
OKC		OKC1		OKC2		OKC3		OKC4		OKC5		OKC6		OKC7		OKC8		OKC9		OKC10	
Epithelial component		BMP4	FGF8	BMP4	FGF8	BMP4	FGF8	BMP4	FGF8	BMP4	FGF8	BMP4	FGF8	BMP4	FGF8	BMP4	FGF8	BMP4	FGF8	BMP4	FGF8
Basal cells	C	−	−	−	−	−	−	−	−	+	−	−	−	−	−	−	+	−	−	−	−
	N	++	++	−	+++	++	+++	+	−	++	−	+++	+++	+	+	+++	+	++	−	+++	++
Parabasal cells	C	−	−	−	−	−	−	−	−	+	−	−	−	−	−	−	−	−	−	−	−
	N	++	++	−	++	++	+++	−	+	++	+	++	+	+	++	+++	++	++	−	+++	++
Mesenchymal component	C	−	−	−	++	−	++	+	−	+	−	+	−	+	+	++	++	+	−	+	−
	N	++	+	−	++	−	++	+	−	+	+	+	−	+	+	++	++	+	−	+	−
AF		AF1		AF2		AF3		AF4		AF5		AF6		AF7		AF8		AF9		AF10	
Epithelial component		BMP4	FGF8	BMP4	FGF8	BMP4	FGF8	BMP4	FGF8	BMP4	FGF8	BMP4	FGF8	BMP4	FGF8	BMP4	FGF8	BMP4	FGF8	BMP4	FGF8
Columnar cells (ameloblast like)	C	+	+	+	++	−	−	++	+	−	+	−	−	−	−	++	−	−	−	+	−
	N	++	++	++	+	+	−	−	+	+	++	−	+	−	−	+	+	+	−	++	−
Ectomesenchymal component	C	−	−	−	−	−	−	−	+	−	−	+	+	−	−	−	+	−	−	−	−
	N	+	−	+	−	−	++	+	−	−	−	−	−	−	−	+	−	+	−	+	++
OM		OM1		OM2		OM3		OM4		OM5		OM6		OM7		OM8		OM9		OM10	
		BMP4	FGF8	BMP4	FGF8	BMP4	FGF8	BMP4	FGF8	BMP4	FGF8	BMP4	FGF8	BMP4	FGF8	BMP4	FGF8	BMP4	FGF8	BMP4	FGF8
Mesenchymal component	C	−	−	−	+	++	+	++	−	−	−	+	−	−	−	+	++	−	+	+	−
	N	++	++	++	+	++	+++	++	+	−	−	++	+	−	+	++	++	+	+	++	+

C: cytoplasm; N: nucleus; Ab: absent.

**Table 3:** Intergroup comparison of expression of BMP4 and FGF8 in odontogenic tumors and odontogenic cyst.

		BMP4				FGF8			
		+++	++	+	−	+++	++	+	−
SMA									
Epithelial component									
Columnar cells (ameloblast like)	C	0	0	1	9	0	0	1	9
	N	3	3	1	3	4	1	5	0
Central cells (stellate reticulum like)	C	0	0	1	9	0	0	0	10
	N	3	3	0	4	2	3	2	3
Mesenchymal component	C	0	1	1	8	0	0	3	7
	N	0	1	2	7	0	0	5	5
OKC									
Epithelial component									
Basal cells	C	0	0	1	9	0	0	1	9
	N	3	4	2	1	3	2	2	3
Parabasal cells	C	0	0	1	9	0	0	0	0
	N	2	5	1	2	1	5	3	1
Mesenchymal component	C	1	6	2	1	0	3	1	6
	N	0	2	6	2	0	3	3	4
AF									
Epithelial component									
Columnar cells (ameloblast like)	C	0	2	3	5	0	1	3	6
	N	0	3	4	3	0	2	4	4
Ectomesenchymal component	C	0	0	1	9	0	0	3	7
	N	0	0	6	4	0	0	2	8
OM									
Mesenchymal component	C	0	2	3	5	0	1	3	6
	N	0	7	1	2	1	2	6	1

C: cytoplasm; N: nucleus.

## Data Availability

The data used to support the findings of this study are available from the corresponding author upon request.
